# Cardiovascular magnetic resonance evaluation for left ventricular diastolic function: analysis of time-volume curve

**DOI:** 10.1186/1532-429X-14-S1-P305

**Published:** 2012-02-01

**Authors:** Takashi Tanimoto, Keizo Kimura, Shingo Ota, Tomizo Masuno, Yuichi Ozaki, Kumiko Hirata, Takashi Kubo, Imanishi Toshio, Takashi Akasaka

**Affiliations:** 1 Cardiovascular Medicine, Wakayama Medical University, Wakayama city, Japan

## Background

Although cine magnetic resonance imaging (MRI) is considered the gold standard for evaluation of left ventricular (LV) volumes, mass, and systolic function, assessment of diastolic filling is still challenging. The aim of this study was to assess the impact of LV mass on diastolic function by time-volume curve obtained from cine MRI in patients with dilated cardiomyopathy (DCM) or hypertrophic cardiomyopathy (HCM) and normal subjects.

## Methods

Cine MRI was performed in 10 healthy controls, 11 HCM patients, and 11 DCM patients. LV end-diastolic volume (EDV), end-systolic volume (ESV), stroke volume (SV), ejection fraction (EF), LV mass, and LVESV/mass ratio were calculated by tracing endocardial and epicardial borders of LV in 8-10 short axis views. Furthermore, to assess the LV diastolic function, the peak filling rate (PFR), time to PFR (TPF), and first third filling fraction (1/3 FF) were calculated from the first derivative curve of the time-volume curve. Temporal resolution was 30 frames per R-R interval.

## Results

Resting LVEF were 54±4% in Control group, 26±9% in DCM group, and 62±6% in HCM group. LVSV were similar in 3 groups. LV mass was greater in DCM and HCM group compared to Control group. TPF of HCM groups was longer than both Control and DCM groups. PFR and 1/3FF were greater in Control group compared to both DCM and HCM groups. LV mass showed significant inverse relationship with PFR (r=-0.65, p=0.0001) and 1/3FF (r= -0.60, p=0.0002).

## Conclusions

LV diastolic parameters can be assessed non-invasively by time-volume curve analysis. LV mass has influence on diastolic filling.

## Funding

None.

